# The B.M.A. and the B.N.A.

**Published:** 1890-09-13

**Authors:** 


### Passing Topics.

### THE B.M.A. AND THE B.N.A

It is reassuring to be informed that "the absence of municipal welcome and hospitality on the occasion of the visit of the British Nurses' Association to this city (Birmingham) for its annual general meeting" is to be satisfactorily explained. When the matter, we are told, was brought before the Mayor, his Worship expressed the greatest regret that, owing to previous and important engagements, it was not in his power to receive or entertain the members of the British Nurses' Association. The fact was that, while on the one hand, the Mayor must needs receive Her Majesty's Judges, who were in attendance for the assizes, on the other, the British Medical Association had also to be considered, and, as is naïvely admitted, that Association preferred a "prior claim upon the Mayor's time and attention."

We are strongly reminded by this incident of the period when we first made formal, and, we fear, somewhat compulsory acquaintance with the Church catechism. To the initial inquiry, "What is your name?" we always felt tempted to answer literally, "N. or M." But just as it was demanded in our case that we should be a little more precise than even a single alternative would allow, so with the British Nurses' Association, the Mayor of Birmingham had no course open to him but to demand whether it was M. or N. who was knocking at the municipal door, and it shows much becoming modesty that, being disappointed of welcome and hospitality, N. is frank enough to admit that M. had the prior claim.

We hope the members of the British Medical Association will be satisfied with this and not suppose for a moment that the British Nurses' Association would have dreamed of displacing them even though the Mayor had displayed a pardonable inclination for the doctrine of "ladies first." We will admit we were not prepared for so much feminine self-abnegation. True, nurses ought to be and often are among the most self-sacrificing of women, and we do not doubt that the members of the British Nurses' Association are not one whit behind their sisters in this and other good qualities, but dealing with them in their collective and therefore insentient capacity, may we not remark that this amusing way of explaining that which needed no explanation is just a little indicative of the spirit certain to animate the body corporate of the British Nursing Association if it ever attain to the actual, as distinguished from the potential, object of its promoters' ambition?

We are quite aware that a number of distinguished members of the faculty have identified themselves with the movement in favour of registration and the erection of nursing into a "profession." But when we consider the absolute exclusiveness of hospital medical men, their intolerance of interference, and, often, almost contemptuous acceptance of lay help, even when it takes so amiable a form as providing money, we are indisposed to believe that the profession of nursing is likely to receive any but a rude reception whenever it raises its head to the level aspired to by certain enthusiasts, mistaken, as we think, in regard to the temper and intentions of those who for the present appear to lend countenance to the movement.

We do not forget the ostentatious rejection of "lay" assistance at the time the British Nursing Association was instituted, but though the promoters bid high for the favour of the medical profession by dissociating themselves from their natural friends and casting themselves at the feet of those they aspired to co-operate with, they will never succeed in altering the conditions upon which their help will be accepted. It is not co-operation, but service which will be demanded at their hands. The blunder of explaining that M. had a prior claim over N. upon the hospitalities of the Mayor of Birmingham was amusing, but a few such errors would be fatal to the professional projects and the somewhat unnatural ambitions the British Nursing Association appears desirous to foster.

Agricola.

